# Predictive Factors of the Burnout Syndrome Occurrence in the Healthcare Workers During the COVID-19 Pandemic

**DOI:** 10.3389/fmed.2022.842457

**Published:** 2022-06-09

**Authors:** Simona Grigorescu, Ana-Maria Cazan, Liliana Rogozea, Dan Ovidiu Grigorescu

**Affiliations:** ^1^Faculty of Medicine, Transilvania University of Braşov, Braşov, Romania; ^2^Emergency Clinical Children Hospital, Braşov, Romania; ^3^Faculty of Psychology and Education Sciences, Transilvania University of Braşov, Braşov, Romania; ^4^Emergency Clinical County Hospital, Braşov, Romania

**Keywords:** burnout syndrome, COVID-19, healthcare workers, work conditions, emotional support, fear of death, meaning of work, public healthcare

## Abstract

The coronavirus disease 2019 (COVID-19) pandemic is probably the most critical epidemiological situation that human civilization has faced in the last few decades. In this context, of all the professional categories involved in the management of patients with COVID-19 are the most likely to develop burnout syndrome. The main objective of this study is to analyze specific predictive factors of the occurrence and development of the burnout syndrome in the healthcare workers involved in the diagnosis and treatment of patients with COVID-19. The study focused on determining factors of the occurrence, development and maintaining the specific burnout syndrome related to the severe acute respiratory syndrome coronavirus 2 (SARS-CoV-2) pandemic infection. The study was conducted on a sample of 959 participants, medical personnel from all the public medical entities in Romania(including 5 hospitals): 122 male and 755 female (82 participants did not declare their gender), with a mean age of 42.29 years (SD = 9.97). The sample included 219 doctors, 477 nurses, 214 auxiliary medical personnel and 49 other types of hospital workers. A cross-sectional design was used. Three predictors of the burnout syndrome were identified: Work conditions, Fear of the consequences (including death) determined by the COVID-19 and Need for emotional support. Meaning of work had a moderating role. Several moderated mediation models were tested. The indirect relationship of Work conditions with burnout *via* Fear of infection was statistically significant; in addition, the indirect effect of Work conditions on burnout through both fear of infection and need for support was statistically significant. The moderation analysis showed that Meaning of work buffer the relationship between Work conditions and Fear of infection. The variance explained by the model including the moderator (30%) was higher than the variance explained by Model 1 (27%), showing that adding the moderating effect of Meaning of work to the relationship of Work conditions with burnout was relevant. The results could be used to design specific interventions to reduce the occurrence of the burnout syndrome in healthcare workers, the implementation of a strategy to motivate employees by highlighting and recognizing the high significance of the work of those in the frontline of the fight against COVID-19.

## Introduction

Human civilization is probably going through the most critical epidemiological situation of this beginning of the millennium, coronavirus disease 2019 (COVID-19) becoming quickly a serious threat to global health and a significant challenge to health systems around the world. The magnitude of the impact of this pandemic is unprecedented, with studies concluding that it could take the world more than a decade to recover medically, socially, psychologically, and economically from COVID-19 (1). In the last 20 years, the world has experienced global public health crises caused by new virus infections, such as the H5N1 subtype of influenza A virus, HIV, the H1N1 subtype of influenza A virus, SARS-CoV1, MERS-CoV, Ebola (2–4). However, the new epidemiological features of COVID-19 represented by the rapid spread of the virus, not only highlighted the lack of preparedness of many governments around the world for such situations (1), but also generated anxiety, depression, panic, the latter effects possibly more harmful in the long run than the virus itself (5). Although recent studies have shown that 80% of people infected with COVID-19 have mild symptoms and a mortality rate of only about 2–3%, the overall mortality from COVID-19 is higher than in Severe acute respiratory syndrome (SARS) and Middle East respiratory syndrome (MERS) infections combined. This characteristic determined the perception of a higher severity of COVID-19 than SARS (6). In this context, one of the most vulnerable professional categories likely to be infected are the healthcare workers. According to the International Council of Nurses, 1,500 nurses have died from COVID-19 in 44 countries up to 28 October 2020 (7). Because COVID-19 can already be considered an epidemic of physical and mental health, in this time of increased stress and uncertainty, it has been more important than ever for healthcare workers to take care of themselves (8).

As stated by the World Health Organization (9), public health workers are likely to experience traumatic experiences, develop burnout syndrome and adopt ineffective coping strategies that can worsen their mental health. First-line healthcare workers, especially employees involved in the diagnosis and treatment of patients with COVID-19, reported high levels of burnout, associated with symptoms including insomnia, depression, and anxiety (10). Medical staff who care for COVID-19 patients experience negative emotions, fear, anxiety, due to fatigue, discomfort, and helplessness related to their high-intensity work (11). The main risk factors that increased nurses' burnout were: younger age (7, 12), decreased social support (13), low family and colleagues readiness to cope with COVID-19 outbreak (7), longer working time in quarantine areas, working in a high-risk environment, working in hospitals with inadequate and insufficient material and human resources, increased workload and lower level of specialized training regarding COVID-19. Based on this, the striking statement was made that every clinician is also a patient and special interventions to promote mental wellbeing in healthcare workers exposed to COVID-19 must be implemented (8, 14, 15).

Studies have showed that healthcare workers are not only under the influence of stress during the epidemic itself, but also long after the initial outbreak has been extinguished, unfortunately, the conditions generating burnout syndrome act long term. In the specific case of COVID-19, this situation highlights the need to identify early burnout factors as predictors for future crises (16).

The main objective of this study is to identify and analyse predictors of the burnout syndrome in the healthcare workers that carried out their activity during the pandemic. We aim to highlight their value and the characteristics specific to the healthcare workers' jobs as predictive factors for the appearance of the burnout syndrome during the pandemic. Predictive factors such as inappropriate work conditions, fear of the consequences (including death) determined by the COVID-19 and need for emotional support, meaning of work and life will be analyzed, taking also in consideration other relevant personal characteristics such as age, gender, being infected during the pandemic. A secondary aim is to propose useful strategies based on the burnout predictors to prevent the occurrence and development of the burnout syndrome, including specific personalized interventions.

### Burnout and Work Characteristics in Healthcare Workers During COVID-19 Pandemic

Burnout represents the consequence of prolonged exposure to stressful working conditions, being characterized by emotional exhaustion, depersonalization, and a diminished sense of personal fulfillment. WHO recently included burnout in the International Classification of Diseases 11th revision as an occupational phenomenon, resulting from chronic workplace stress unsuccessfully managed (9). The burnout syndrome is listed in ICD-10 in the Z 73.0 category but not yet mentioned in the 5th edition of the Diagnostic and Statistical Manual of Mental Disorders.

In healthcare professionals, burnout has a negative impact both on a personal level, and on the quality of patient care, generating secondarily the occurrence of medical errors, increased risk of malpractice and alcohol or drug use (17). The physician burnout is augmented by work conditions (excessive workloads, working hours), personal characteristics (work-life imbalance, inadequate support, sleep deprivation), and organization factors (workload expectations, interpersonal communication, negative leadership) (15, 18, 19). Even before this pandemic, the excessive workload, the tasks assigned in addition to the clinical ones, the ambiguity of the attributions or the multiple deficiencies of the medical system generated high levels of burnout in the healthcare workers (20). The current COVID-19 pandemic has disrupted the global health system, creating additional stress for clinicians (21, 22).

COVID-19 is characterized by multiple stressors acting on healthcare providers, including the risk of infection, social isolation and economic consequences (23). The practice of medicine has changed in the COVID-19 period, with decreasing outpatient revenue, reductions in salary and benefits, and increased use of telemedicine, with its specific effect on the doctor-patient relationship (24). Limitation of testing, uncertainty caused by the permanent change in WHO recommendations, redistribution of physicians in less familiar but critical clinical areas were other causes of anxiety. Doctors faced ethical dilemmas, they felt that their medical oaths were tested due to the particularity of caring for infected patients (22).

In developing countries, where the healthcare system was already either overloaded or precarious, an additional stress appeared, generated by the hospital's inadequate supply of materials for hygiene and a significant lack of personal protective equipment, determining the highest risk of transmitting the COVID-19 infection. Taking care of sick colleagues during the pandemic has increased the healthcare workers' anxiety concerning their competence and abilities, making them even more mentally vulnerable (5). On the contrary, healthcare workers who were not involved in the direct care of patients with COVID-19 and therefore remained at home indefinitely, experienced feelings of isolation and worthlessness in terms of their ability to effectively contribute to the current crisis, which in some cases led to existential questions concerning the meaning of work (25).

Being directly involved in the care of infected or suspected of suffering from COVID-19 patients, social isolation and being separated from their family determined high levels of stress. Also, the healthcare workers who returned home to their family every day had an increased risk of burnout due to the fear of transmitting the disease, especially if their family's included elderly or people with chronic diseases at higher risk of complications. The lack of safe treatment protocols and vaccines in the early stages of the pandemic, the risk of disease transmission and infection, even in the asymptomatic form of the disease, the lack of training in infection control procedures determined a phenomenon of resignations in the beginning of the pandemic. The occurrence of deaths among healthcare workers led to an increase in their stress level with repercussions on physical health represented by cardiac pathology in the form of arrhythmias or myocardial infarction (6, 26).

Changing shifts, work schedule and type of current activity, the technique of putting on and taking off the protective equipment represented a novelty factor with a stressful effect. Wearing protective equipment leads to a depersonalization of the activity, both in relation to patients and colleagues with whom the activity is carried out. The impossibility of reading facial expressions and the lack of interpersonal interactions decreased the possibility of socialization and mutual encouragement. Moreover, wearing heavy protective clothing and N95 masks made it much more difficult to perform certain interventions or medical procedures than under normal conditions (26, 27). Other burnout factors are lack of control over procedures, ineffective infection control measures, false notion of safety measures, poor communication and unclear directives, lack of training and emotional support, inadequate personal protective equipment and the perception of imminent fatality (16).

### Fear of COVID-19 and Burnout

Older age, the existence of elderly or of people with chronic illnesses in the family were risk factors for burnout and were associated with intense fear of COVID-19 (10, 27, 28). Also, first-line healthcare workers who had close contact with infected patients, including those working in the disease respiratory wards, emergency rooms or infectious disease departments showed a high level of fear of COVID-19 (5, 29).

The healthcare workers' fear to become infected was a significant factor that influenced the quality of patient care (30). Because of the high contagiousness, the biggest fear generating burnout was the infection of family members (21, 31). As more and more deaths were reported among healthcare workers, their fear increased (32). Workload of treating suspected COVID-19 patients, fear of COVID-19 infection, anxiety, and depression were significant predictors of burnout hospital health workers during the COVID-19 outbreak (33).

### Need for Support and Burnout

The healthcare workers' intense fear of illness was also fuelled by the doubt that the institution would support them if they were infected, by the lack of access to childcare facilities during the quarantine, blocking of access to home and by the lack of accurate information about the disease (34). Social or emotional support from colleagues, managers, friends and family is considered to be extremely important for the healthcare workers to deal effectively with workplace stressors (35). Previous studies identified the positive effects of social support on the healthcare professionals' mental health, work involvement and job satisfaction (30). Adequate social support was also considered vital to help public health employees effectively manage stressful events, including emergencies, disasters, pandemics (36, 37).

Organization support policies and measures supporting healthcare professionals' mental, psychological, and emotional health could create a safe work environment and represent effective methods to control COVID-19 anxiety (38). Studies showed that organizational support in medical settings is positively associated with job satisfaction, performance and patient satisfaction (37) being considered a protective factor against burnout and anxiety. Other studies also showed that higher levels of emotional support are associated with higher healthcare workers self-efficacy, better sleep quality and low levels of stress and anxiety (39), healthcare professionals being exposed to a higher risk of experiencing mental health problems and deterioration of the quality of their professional life (40).

### Meaning of Work, Work Conditions, and Burnout

Working life changed dramatically during the COVID-19 pandemic, the crisis changed employees' lifestyle, expectations, priorities, the meaning of work and life (41). Purpose and significance of life in general, as component of meaning (42) were affected by the pandemic, higher levels of meaning in life and work being related to lower anxiety and emotional distress during crisis (43). Having purpose in life is essential to wellbeing and health, and more specifically meaning of work influences important outcomes such as work motivation, work behavior, engagement, job satisfaction, empowerment, burnout and work performance (44).

Research showed that the perception of healthcare professionals on the importance of meaning of life as part of their professional life may work as a buffer against stress reactions to the pandemic, being considered a protective factor against burnout (45) in healthcare workers (46). On the other hand, meaningful work can be a risk factor for burnout leading employees to continue increasing their efforts beyond their limits. However, the negative associations between meaningful work and burnout in physicians and nurses were more frequently highlighted (47). The degree to which people were affected by the pandemic differs from person to person and it also depends on pre-existing vulnerabilities, anxiety, stress vulnerability, empathy, but also on work conditions and the amount of social and emotional support received (48, 49). Therefore, we hypothesize that meaning of work will have a buffering role in the relationship of work conditions with burnout. In this context, our study will focus on emphasizing the determining factors of the occurrence, development and maintaining the specific burnout syndrome related to the severe acute respiratory syndrome coronavirus 2 (SARS-CoV-2) pandemic infection. We proposed the following hypothesis:

H1. Fear of infection or death by COVID-19 mediates the relationship between Burnout and Work conditions.
H2. Need for emotional support mediates the relationship between Burnout and Work conditions.
H3. Both fear of infection or death by COVID-19 and Need for emotional support mediate the relationship between Burnout and Work conditions.
H4. Meaning of work moderates the relationship of Work conditions with Fear of infection or death by COVID-19, a high level of Meaning of work reducing the negative effect of inappropriate Work conditions on Fear of COVID-19.
H5. Meaning of work moderates the relationship of Work conditions with Need for emotional support, a high Meaning of work reducing the negative effect of inappropriate Work conditions on Need for emotional support.
H6. The serial indirect relationships of burnout with Work conditions *via* Fear of infection or death by COVID-19 and Need for emotional support are moderated (attenuated) by Meaning of work.

## Methods

### Participants

The sample included 959 participants, m (50) edical personnel from all the public medical entities (including 5 hospitals) in Brasov, Romania: 122 male and 755 female (82 participants did not declare their gender), with a mean age of 42.29 years (*SD* = 9.97, *X*_*min*_ = 20, *X*_*max*_= 69). Missing data for gender were handled using the method of median imputation; since the sample included more females than males, the imputation resulted in assigning female to all observations with missing gender. The decision was also based on the fact that the majority of missing data for gender belonged to nurses (as professional category) and in Romania most of the nurses are females. Convenience sampling was used for this study. The sample included 219 doctors, 477 nurses, 214 auxiliary medical personnel and 49 other types of hospital workers. Regarding their working place, 801 participants are employees in Hospitals, 44 in Emergency Units, 39 in Ambulance, 56 in Outpatient Clinics, 25 are General Practitioners. Forty-seven (47) participants were tested positive for SARS-COV-19 at the moment of the study. Concerning the availability of special equipment for the type of treated patients, most of the participants declared that all the necessary conditions were met: always (*N* = 624), most of the time (*N* = 260), seldom (*N* = 48), never (*N* = 16). Concerning the duration of healthcare workers interaction with the patients (mean of weeks) 505 participants declared they were direct contacts with Non-COVID 19 patients (*M* = 5.35, *SD* = 5.5, *X*_*min*_ < less than a week, *X*_*max*_ = 14), 584 were direct contacts with COVID-19 patients (*M* = 6.14, *SD* = 4.91, *X*_*min*_ < less than a week, *X*_*max*_= 14) and 727 were direct contacts with COVID-19 suspect patients (*M* = 3.66, *SD* = 4.56, *X*_*min*_ < less than a week, *X*_*max*_= 13).

The research was conducted for 4 months of the lockdown period in Romania. The research followed the Helsinki Declaration and the data protection regulation of Italy (Legislative Decree No. 196/2003). Participation was voluntary and not rewarded. Data collection and analysis were anonymous. A cover letter attached to the questionnaire provided information about the study aims, guarantees about anonymity, voluntary participation, data treatment and instructions for filling out the questionnaire. By agreeing to fill out the questionnaire, participants provided their informed consent.

### Measures

- Burnout was assessed through the Copenhagen Burnout Inventory—the Romanian version (51, 52) containing 18 items on a 5-point Likert scale, grouped in three dimensions. The personal burnout refers to the personal exhaustion, the degree of physical and mental fatigue (7 items, example of items: *How often do you feel tired? How often are you emotionally exhausted?)*. The work-related burnout represents the perceived physical and psychological fatigue related to the professional activity (7 items, *Do you feel worn out at the end of the working day*? *Do you feel that every working hour is tiring for you?*). The patient-related burnout refers to the physical and mental fatigue and exhaustion perceived by the affected person as being related to the work with clients/patients (6 items, *Do you find it hard to work with clients?, Does it drain your energy to work with clients?*).
- Meaning of work (MoW) was measured with a three-item scale from the Second version of the Copenhagen Psychosocial Questionnaire (53): *Is your work meaningful? Do you feel that the work you do is important? Do you feel motivated and involved in your work?* The three items are measured on 5-point Likert scale.
- Meaning of life was measured with a three-item scale adapted from the Meaning in Life Scale (54). The items were measured on a 4-point Likert scale (*I have discovered a clear meaning of life, I have discovered new directions in life that satisfy me, I know exactly what gives meaning to my life)*. The Exploratory Factor Analysis using Varimax rotation revealed a one-factor solution, covering 74% of the variance.
- The need for emotional support (NES) and perceived emotional support received as an employee were measured with 12 items on a 4-point item scale, 6 items for need for support and 6 items for the perceived emotional support received as an employee. The Varimax Exploratory Factor Analysis revealed a two-factor solution covering 58.79% of the total variance. The first factor, need for emotional support has an eigenvalue of 5.5 and it covers 32.29% of the variance and the second factor, perceived emotional support received, has an eigenvalue of 1.54 and covers 25.85 of the variance. Example of items*: To what extent did you feel the need for emotional support / did you receive emotional support during the COVID-19 period from: family, friends, acquaintances, colleagues, superiors?*
- The COVID-19 work context was assessed with 13 items measured on a 4-point Likert scale. The Varimax Exploratory Factor Analysis revealed a two-factor solution covering 59.89% of the total variance. The first factor labeled *Work conditions* (WOC) included items referring to inappropriate work conditions (WOCI) context such as inadequate communications between superiors and subordinates, dysfunctional relationships, insufficient stuff and equipment; the factor has an eigenvalue of 6.14 and it covers 36.94% of the variance. The second factor was labeled *Fear of consequences* (*including death) determined by the COVID-19* (FID) and included items such as: *There is a high probability of being infected, I am afraid of a possible serious evolution of illness after SARS-CoV-2 infection, etc*. The factor has an eigenvalue of 1.64 and covers 22.95% of the variance.
- Emotional and somatic symptoms were measured with two subscales adapted from the Second version of the Copenhagen Psychosocial Questionnaire (53): 6 items for emotional symptoms (*During last month, how often have you felt stressed, tensed, irritated, exhausted, sad, unmotivated?)* and 5 items for somatic symptoms (*During last month, how often have you had a headache, palpitations, stomach-ache, tension in various muscles, mental concentration difficulties?*). All items were measured on a five-point Likert scale.
- Socio-demographic data were assessed with several questions regarding age, gender, seniority, type of employment, type of healthcare organization, category of patients treated during the COVID-19 pandemic period. Items about their status and health condition as a COVID-19 patient were also added: *Where you infected?, What symptoms did you have?, What was the duration of hospitalization (in number of weeks)*.

Cronbach's Alpha values for all the scales are presented in Table 1, showing good reliability. To estimate the construct validity of the scales included in Study 1, we conducted a confirmatory factor analysis. Several factor structures were analyzed, the five-factor model having the best fit indices. A seven-factor structure was tested (three factors for burnout—personal, work, client related burnout, meaning of work, fear of COVID consequences, need for support): *CMIN/df* = 3.90, *p* < 0.001, *RMSEA* = 0.05, *CFI*= 0.90, *AIC* = 889474.29. The five-factor model including a total burnout dimension showed better fit indices: *CMIN/df* = 3.56, *p* < 0.001, *RMSEA* = 0.05, *CFI*= 0.92, *AIC* = 88734.23.

**Table 1:** Descriptive and reliability indicators, pearson coefficient correlations.

	***Sk***	***Ku***	***M***	***SD***	**1**	**2**	**3**	**4**	**5**	**6**	**7**	**8**	**9**	**10**	**11**	**12**
1 Personal burnout	0.32	−0.33	2.85	0.87	(0.90)											
2 Work burnout	0.36	−0.51	2.62	0.89	0.75***	(0.88)										
3 Patients related burnout	0.90	0.53	1.93	0.90	0.54***	0.60***	(0.93)									
4 Burnout total	0.59	−0.08	2.47	0.77	0.87***	0.90***	0.82***	(0.93)								
5 Need for support	0.09	−0.50	2.50	0.79	0.30***	0.25***	0.21***	0.29***	(0.88)							
6 Received emotional support	0.11	0.06	2.58	0.67	0.07*	0.04	−0.01	0.04	0.58***	(0.81)						
7 Emotional symptoms	0.21	−0.57	2.92	1.00	0.57***	0.56***	0.35***	0.57***	0.36***	0.13***	(0.87)					
8 Somatic symptoms	0.78	0.16	2.05	0.85	0.56***	0.52***	0.41***	0.57***	0.29***	0.09**	0.58***	(0.82)				
9 Meaning of work	−0.86	0.79	4.05	0.91	−0.15***	−0.18***	−0.20***	−0.20***	0.02	0.11****	−0.12***	−0.10**	(0.80)			
10 Meaning of life	0.12	−0.93	2.42	0.92	0.16***	0.14***	0.12***	0.16***	0.29***	0.18***	0.17***	0.20***	0.13***	(0.82)		
11 Fear of COVID	−0.13	−0.55	2.73	0.75	0.43***	0.43***	0.301**	0.44***	0.43***	0.20***	0.50***	0.45***	−0.01	0.29***	(0.80)	
12 Work inappropriate conditions	0.26	−0.65	2.30	0.81	0.41***	0.43***	0.33***	0.45***	0.29***	0.04	0.49***	0.40***	−0.16***	0.16***	0.56***	(0.90)

***p < 0.001, **p < 0.01, *p < 0.05, N = 959. Cronbach's Alpha was listed on the main diagonal.

### Data Analysis

The preliminary power analysis and sample size calculation were run using the Monte Carlo Power Analysis for Indirect Effects (55). For an observed power of 0.80, mor serial mediation model, a 95% confidence interval and 5,000 samples, we estimated a sample size of ~600 participants required % for detecting the hypothesized indirect effects. Our sample included 959 participants which could ensure a higher power (>0.95).

We used a cross-sectional design to explore the associations between burnout and work conditions, fear of the consequences (including death) determined by the COVID-19, need for emotional support, meaning of work, meaning of life, etc. To test the main hypothesis, a serial multiple mediator model (Model 6 in Process 3.0) and then a moderated serial multiple mediator model (Model 85 in process 3.0) (56) were utilized. The statistical significance of the index of moderated mediation, and the moderated-mediation and moderated serial-mediation effects were assessed by interpreting the 95% bias-corrected confidence interval (5,000 samples). Mean centering was used as recommended in the literature on moderation analysis, mean centering reducing multicollinearity between the product and the constituent terms of the interaction but without any effect on the hypotheses testing (56).

## Results

### Preliminary Analyses—Bivariate Associations Between the Study Variables

The Pearson correlations between burnout dimensions, meaning of life, meaning of work (MoW), need for emotional support (NES), fear of the consequences (including death) determined by COVID-19 (FID) and work inappropriate conditions (WOCI) showed significant moderate associations between burnout and all the other variables, except for received emotional support (the Pearson correlation coefficients being lower than 0.07 and significant) and meaning of life and work, which showed small associations (lower than 0.20 but highly significant, with significance levels lower than 0.001). Meaning of work and burnout are negatively, while meaning of life and burnout are positively correlated. Fear of COVID-19 consequences is moderately associated with all the burnout dimensions showing that this factor could be an important predictor of burnout; work conditions and fear of COVID-19 consequences are also positively corelated, which means that fear of COVID-19 consequences could be a mediator between work conditions and burnout. Need for emotional support is also positively associated both with work conditions and burnout, leading to a further hypothesis of a possible mediation role of Need for emotional support (Table 1). The analysis of the correlation coefficients between burnout and the other dimensions allowed us to identify the most relevant predictors of burnout to be used in the mediation analysis.

### Hypothesis Testing

**Figure 1:**
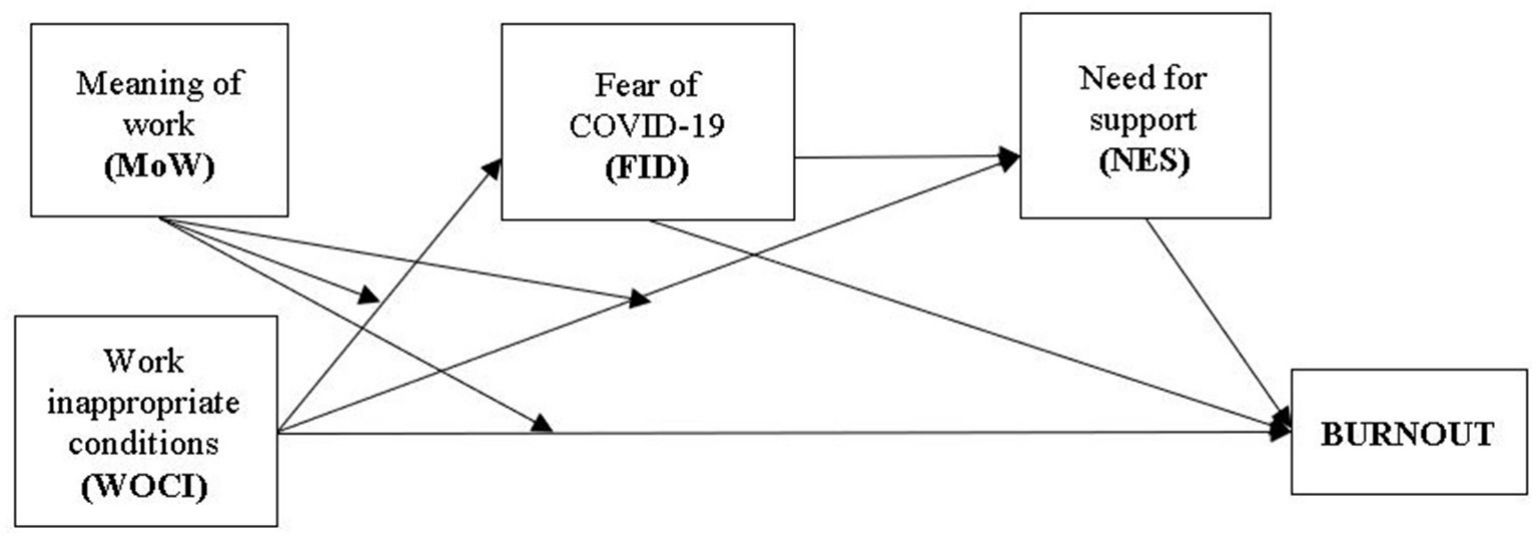
The conceptual model.

Several moderated mediation models were tested, two models being significant: Model 1, the serial multiple mediator model (Model 6 in Process 3.0) and Model 2, the moderated serial multiple mediator model (Model 85 in Process 3.0) (56). Model two was the most appropriate for explaining the relationships between the study variables. This model allowed us to test (a) the specific indirect effect through fear of the consequences (including death) determined by the COVID-19, (b) the specific indirect effect through need for emotional support, and (c) the indirect effect through both fear of the consequences (including death) determined by COVID-19 and the need for emotional support as serial relationship, thus considering the positive relationship between the two variables. Age, Gender and Being infected were added as control variables. In our model, Meaning of work is the moderator (three moderation effects are analyzed: the interaction of (1) Meaning of work and Work conditions on Fear of COVID-19 consequences, (2) on Need for support and on (3) Burnout. The fear of COVID-19 and Need for emotional support are serial mediators, meaning that Work inappropriate conditions could increase Fear of COVID-19 consequences which in turn could increase Need for emotional support, leading to higher burnout (Figure 1).

#### Mediation Effects of Fear of COVID-19 Consequences and Need for Emotional Support

Table 2 presents the results of the serial mediation analyses. Model 1 includes a three-path mediated effect: the first path refers to the relationship between Work conditions and Burnout, the Fear of COVID-19 being the mediator (H1); the second path included the relationship between Work conditions and Burnout, the mediator being the Need for emotional support (H2); the third path included the relationship between Work conditions and Burnout, mediated by both the Fear of COVID-19 and the Need for emotional support (H3). The indirect and serial indirect relationships are analyzed in Table 2. In line with H1, the indirect relationship of work conditions with burnout *via* fear of infection was statistically significant. Hypotheses 2 was not supported, the indirect effect of work conditions through need for support being not significant. However, the serial mediation analysis, the indirect effect of work conditions on burnout through both fear of infection and need for support was statistically significant. Hypothesis 3 was supported.

**Table 2:** Path estimates (unstandardized coefficients) for the mediation analyses.

	**Path estimates**	**Confidence intervals**		***R* ^2^**	**Sig**
		**LLCI**	**ULCI**		
**Model 1: Serial multiple mediator model**					
Predicting fear of COVID-19				0.33	<0.001
Constant	−0.28	−0.41	−0.11		0.001
Work inappropriate conditions	0.52	0.47	0.57		<0.001
Covariate: age	0.006	0.002	0.010		0.001
Covariate: being infected or not	−0.06	−0.25	0.12		0.507
Covariate: gender	0.15	0.03	0.27		<0.001
Predicting need for emotional support				0.18	<0.001
Constant	−0.14	−0.34	0.06		0.173
Work inappropriate conditions	0.08	0.01	0.15		0.020
Fear of COVID-19	0.39	0.30	0.45		<0.001
Covariate: age	0.003	−0.001	0.008		0.173
Covariate: being infected or not	0.04	−0.17	0.27		0.664
Covariate: gender	0.11	−0.01	0.25		0.092
Predicting burnout				0.27	<0.001
Constant	−0.17	−0.36	0.01		0.068
Work inappropriate conditions	0.27	0.21	0.34		<0.001
Fear of COVID-19	0.24	0.17	0.32		<0.001
Need for emotional support	0.10	0.04	0.16		0.001
Indirect WOCI effect through	0.13	0.09	0.17		<0.001
Indirect WOCI effect through	0.008	0.001	0.01		0.034
Indirect WOCI effect through FID and NES	0.02	0.009	0.03		<0.010
Covariate: age	0.004	0.001	0.009		0.054
Covariate: being infected or not	−0.01	−0.22	0.19		0.192
Covariate: gender	−0.05	−0.17	0.07		0.381
**Model 2: Moderated serial multiple mediator**					
Predicting fear of COVID-19				0.34	<0.001
Constant	−0.25	−0.42	−0.79		0.004
Work inappropriate conditions	0.53	0.48	0.58		<0.001
Meaning of work	0.08	0.03	0.12		<0.001
WOCI x MoW	−0.05	−0.10	−0.01		0.010
Covariate: age	0.005	0.001	0.009		0.005
Covariate: being infected or not	−0.09	−0.28	0.09		0.343
Covariate: gender	0.16	0.04	0.27		0.005
Predicting need for emotional support				0.18	<0.001
Constant	−0.12	−0.32	0.08		0.236
Work inappropriate conditions	0.09	0.03	0.16		0.010
Fear of COVID-19	0.37	0.30	0.45		<0.001
Meaning of work	0.03	−0.01	0.08		0.170
WOCI × MoW	0.005	−0.04	0.05		0.863
Covariate: age	0.002	−0.001	0.007		0.233
Covariate: being infected or not	0.04	−0.18	0.26		0.707
Covariate: gender	0.11	−0.02	0.25		0.09
Predicting burnout				0.30	<0.001
Constant	−0.24	−0.43	−0.05		0.016
Work inappropriate conditions	0.23	0.17	0.30		<0.001
Fear of COVID-19	0.26	0.19	0.34		<0.001
Need for emotional support	0.11	0.05	0.17		<0.001
Meaning of work	−0.15	−0.19	−0.10		<0.001
WOCI × MoW	−0.005	−0.05	0.04		0.884
Covariate: age	0.005	0.001	0.01		0.007
Covariate: being infected or not	0.01	−0.19	0.0.21		0.897
Covariate: gender	−0.05	−0.18	0.0 6		0.370

#### Moderating Effects of Meaning of Work

**Figure 2:**
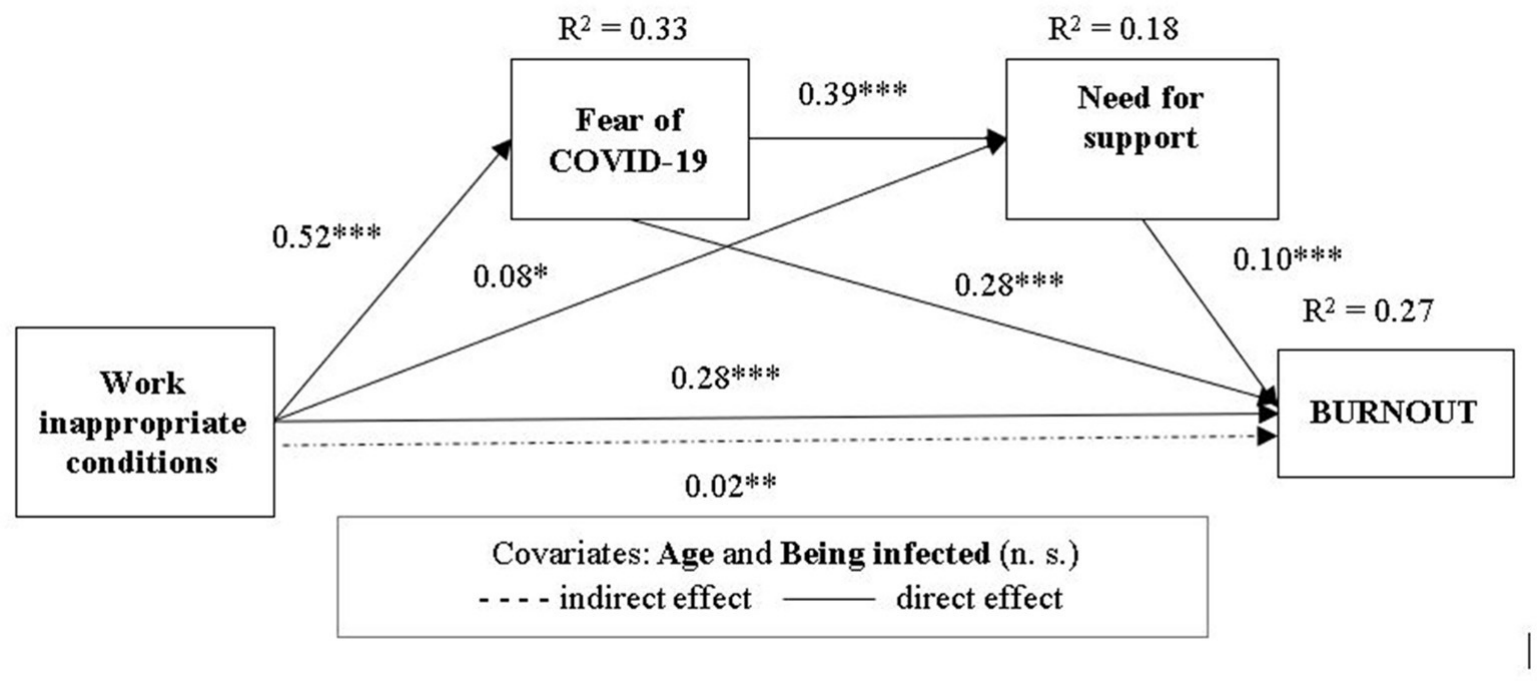
Model—mediational model of relationship between Work inappropriate conditions and Burnout through Fear of COVID-19 consequences and Need for emotional support.

**Figure 3:**
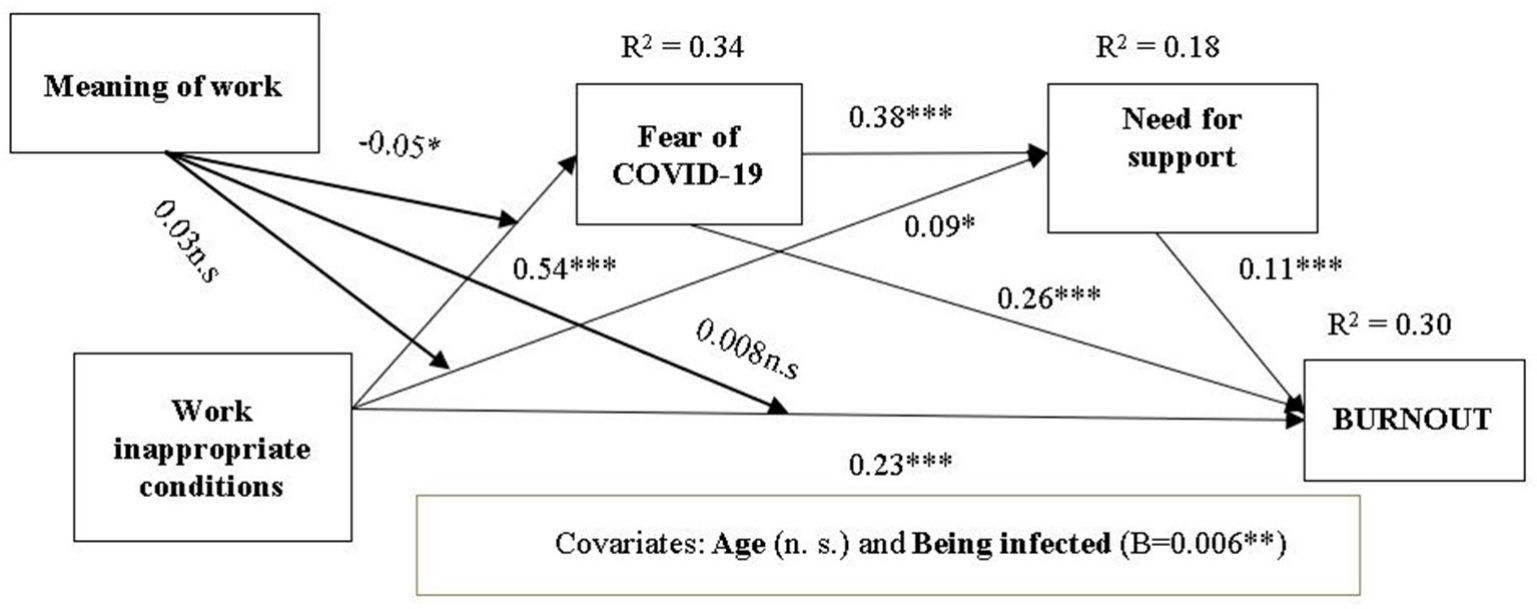
Model 2 moderated serial multiple mediator of relationship between Work conditions and burnout through Fear of COVID-19 consequences and Need for emotional support, moderated by the Meaning of work.

Subsequently, in Model 2, a moderated mediation model was tested to examine the moderating effects of Meaning of work. In this model, the indirect effect of Work conditions on Burnout *via* Fear of infection was moderated by Meaning of work (H4). No evidence of a significant interaction was found for the indirect effect *via* Need for emotional support (H5). As for hypothesis 6 (H6), the index of moderated mediation was significant but in the absence of significant total interaction of Work conditions and Meaning of work (Tables 2, 3). Thus, only hypothesis H4 was supported. However, the variance explained by Model 2 (30%) was higher than the variance explained by Model 1 (27%), showing that adding the moderating effect of Meaning of work to the relationship of Work conditions with Burnout was relevant (Figures 2, 3). Thirty-three percent of the variance of Fear of COVID-19 consequences are explained by Work conditions, while 18% of the variance of Need for emotional support are explained by the effects of Work conditions and Fear of COVID-19.

**Table 3:** Indices of moderated mediation and specific indirect and serial indirect relationships at low, average, and high levels of meaning of work.

	**Index of moderated mediation**	**LLCI**	**ULCI**	**MoW**		
				**Low (−1SD)**	**Average**	**High (+1SD)**
WOCI → FID → Burnout	−0.015	−0.027	−0.004	0.153	0.140	0.128
WOCI → NES → Burnout	0.001	−0.006	0.007	0.010	0.010	0.011
WOCI → FID → NES → Burnout	−0.002	−0.005	−0.0005	0.025	0.023	0.020

To test our predictions, we also interpreted the interaction terms and computed the indices of moderated mediation for the serial indirect relationships. In line with our hypothesis, the interaction terms that predicted Burnout were significant for Fear of infection and the serial path of fear of infection and need for support (Table 3), with the confidence intervals not including the value 0. The index of moderated mediation showed that the serial indirect relationships of Work inappropriate conditions with burnout, Fear of infection and Need for support was moderated by Meaning of work.

We hypothesized that Meaning of work would buffer the relationship between Work conditions and Fear of infection. The interaction between Work inappropriate conditions (WOCI) and Meaning of work (MoW) is negative, meaning that the increase of the MoW would have a decreasing role on the effect of the WOCI on the Fear of infection (FID). The path estimates showed that the effect of WOCI on FID is positive, meaning that individuals working under highly inappropriate conditions will perceive a high fear of infection and of its negative consequences. Given the significant negative interaction, our results showed that the effect of Work inappropriate conditions on Fear of COVID-19 consequences will be less positive when meaning of work increases. Inappropriate work conditions have impact on Fear of COVID, this effect being higher when Meaning of work is low. The moderation analysis showed that inappropriate Work conditions explain the Fear of infection, but high Meaning of work could diminish the negative effect that work conditions have on fear of infection; thus, medical personnel with a strong sense of their meaningful work will not emphasize the inappropriate work conditions to manage their fear of a possible infection and negative consequences, their meaning of work being more relevant for their professional identity than the external factors, namely work conditions.

## Discussion

### Associations Between the Study Variables and the Burnout Syndrome Occurrence

In our study, the strongest associations with burnout were found for Work conditions, Fear of the consequences (including death) determined by the COVID-19, Need for emotional support, Meaning of work and Meaning of life. Work conditions, Fear of COVID-19 and Need for emotional support were the most efficient predictors of burnout. Meaning of life was not included as predictor because its insignificant effective role at the organizational level, being more relevant at the individual level, especially since it has as determinants each respondent's cultural and philosophical beliefs. The occurrence of the burnout syndrome does not correlate with the perception of the received emotional support. The most probable explanation is that, in the study group, the emotional support was not only not received from those who should have offered it, but the participants did not even have such an expectation, for reasons of personal experience in relation to the functioning of the health system institutions in Romania.

The direct influence of Need for emotional support, Inappropriate work conditions, Fear of the consequences determined by the COVID-19 on the development of the burnout syndrome is moderated by Meaning of work, decreasing their effect on the burnout. This indirect inhibitory effect on burnout is associated with the direct action of the Meaning of work on the occurrence of burnout. Characterized by a strong meaning of work, the healthcare workers are more protected from the negative effects of burnout and the arising burnout itself. The relationships between burnout and Work conditions, Fear of COVID-19 and Need for emotional support showed that, among the predictors of burnout, the most important ones are, almost equal in terms of effectiveness, Work inappropriate conditions and Fear of the consequences (including death) determined by the COVID-19 (24).

### Predictors of the Burnout Syndrome

#### Direct Predictors of Burnout

The occurrence and development of burnout syndrome has been statistically demonstrated to have as direct predictors all three variables taken into analysis:

a) Work conditions are a strong direct predictor of the burnout syndrome in the epidemiological context of the SARS-CoV-2 pandemic, the explanation being that poor working conditions induce in any situation to the healthcare workers the occurrence of the exhaustion syndrome (50), the occurrence of the exhaustion syndrome in healthcare workers in any situation. As a result, the development of burnout syndrome will be favored in the much more demanding context of further overlap of the consequences of the pandemic.
b) Fear of the consequences (including death) determined by the COVID-19 is a significant direct predictor (57). As highly specific element in the studied context, the fear of disease and death strongly influences the occurrence of the emotional exhaustion of healthcare workers involved in the medical management of COVID-19 (58).
c) Even the Need for emotional support is also a direct predictor, its predictive weight is lower. Although the emotional support could minimize burnout, the participants did not actually consider it an effective mechanism, in the context of the lack of emotional support they receive from their leaders and managers. This perception is explicable in the context of the poor managerial attitude constantly proven over the last decades in the Romanian public health system (59).

#### Relationships Between Work Conditions Inappropriateness, Fear of the Consequences (Including Death) Determined by the COVID-19 and Need for Emotional Support

a) Work conditions are a strong predictor of fear of COVID-19; inappropriate work conditions expose healthcare workers to a higher risk of disease and to the risk of serious evolution (including death), which generates a specific and explicable fear of COVID-19 (60).
b) Despite their important potential, work conditions do not have much influence on the need for emotional support. The explanation could be that the institutional leaders and managers of the Romanian pubic national health system did not care in the long run about optimizing working conditions and ignored the healthcare professionals' needs (61). In the long-term context, it is more than likely that the latter have detached from their expectations of being emotionally supported within the system.
c) Fear of the consequences (including death) determined by the COVID-19 is a significant predictor for the need for emotional support, more a subject fears the disease and its consequences, the greater his need for emotional support is (50), in the sense of psychological support and balance as a member of the community of healthcare providers.

#### Indirect Effects of the Work Conditions and Need for Emotional Support on Burnout

a) Work conditions have not only direct, but also indirect effects on burnout, mediated by the Fear of COVID-19. Work conditions as predictor acts dually on the burnout syndrome, both directly and indirectly, through the Fear of the COVID-19 consequences, generating burnout and maintaining a strong sense of fear. This fear is manifested both toward the possibility of getting sick and the further negative consequences of the disease, including death (62).
b) The indirect effect of Work conditions as a predictor of burnout through the Need for emotional support is less significant; the Need for support, as a direct predictor of the burnout syndrome, proved to be a factor of little importance, but the median analysis showed that it could act as a blocking mechanism, inhibiting the effect of Work conditions on the burnout syndrome. A possible explanation for this slowdown in the transmission of the burnout effects can be the real lack of respondents' need for emotional support, in the absence of any pathways to manifest this need, canceled a long time ago through the absence of institutional support (63).
c) The indirect effect of Work conditions as a predictor of the burnout syndrome, through the simultaneous association between the Fear of COVID-19 and the Need for support was significant. Although the values are significantly higher than those in the inter-relationship Inappropriate work conditions—Need for emotional support—Burnout, they are much lower than in the inter-relationship Inappropriate work conditions—Fear of the consequences determined by the COVID-19—Burnout. This situation emphasizes once again the mediating role of the Need for emotional support. The more neglected over time the healthcare workers' emotional support needs are, the higher the burnout syndrome is, precisely because of the healthcare workers' lack of expectation of emotional support from the system and the emergence of the feeling that each member of the medical community is forced to rely only on their own strengths and adjustment possibilities (64).
d) The indirect inhibitory effect of the Need for emotional support, the need for support is a mediator between the Fear of COVID-19 and Burnout: the occurrence of the burnout syndrome is higher if the Need for emotional support is associated simultaneously with the inappropriate Work conditions and Fear of COVID-19. In other words, work conditions have, at the same time, both a direct effect on the occurrence of the burnout syndrome and an indirect effect through the fear of COVID-19. In turn, the indirect effect of the need for emotional support acts as an inhibitory mechanism: the Need for emotional support reduces the negative effect of Work conditions through Fear of the COVID-19 consequences (63).

#### Meaning of Work, as a Moderating Factor

Additional relationships explaining the burnout syndrome were revealed by meaning of work as a moderator.

a) As a direct predictor, meaning of work showed negative effects on burnout. The more the participants emphasize in their personal philosophy the importance of their role in ensuring public health, the more blocked the effects of the other predictors are. In other words, the more significant the role of the healthcare workers is felt as concerning their relation to the patients, the less influenced they are by the poor quality of the working conditions as a determining factor of the burnout syndrome (46).
b) The role of Meaning of work as a direct predictor of Fear of the consequences (including death) determined by the COVID-19 proved to be weak but inhibitory. The explanation is identical to the one above, with the only difference that fear of the consequences (including death) determined by the COVID-19 is more important given that the participants assume a defining role in the care and treatment of patients with SARS CoV-2 infection (65). Work inappropriate conditions are a strong predictor of the Fear of COVID-19, but this relationship was moderated by the Meaning of work. Thus, the presence of a high Meaning of work is reducing the negative effect of Work inappropriate conditions on Fear of infection. The moderating role of the meaning of work in the relationship between the fear of the consequences (including death) and burnout lies in the prevalence of the philosophical perception of a subject's role in the lives of others on the same subject's fear of illness or death (27). Previous studies also showed that commitment, work motivation and resilience could help healthcare professionals find strategies to cope with difficulties and to consider them professional challenges, reducing the negative effects of stress (66, 67).
c) Meaning of work was not a direct predictor of the Need for emotional support. When a person is facing a threat (in this case their power being determined by the perception of their own value in the community), the need for emotional support disappears as an element of personal balance. The role of meaning of work could be determined by the perception of the role that everyone has in the life of others and by the philosophical perception on the fear of illness or death (43).

## Conclusions

The strongest associations with the burnout syndrome arising and development were found for Work conditions, Fear of the consequences (including death) determined by the COVID-19, Need for emotional support, Meaning of work and Meaning of life. The most efficient predictors of burnout were the Work conditions, the Fear of COVID-19 and the Need for emotional support, the occurrence and development of the burnout syndrome during the COVID-19 pandemic being predicted to a high extent by the inappropriate Work conditions and the Fear of the consequences (including death) determined by the COVID-19 and to a lower extent by the Need for emotional support. All these three dimensions were demonstrated to be direct determinants of the level of burnout experienced by the healthcare workers. More than that, we put into evidence some additional relationships between Work conditions, Fear of the consequences (including death) determined by the COVID-19 and Need for emotional support which provide a supplementary influence of the burnout syndrome development: inappropriate Work conditions are a strong predictor also for Fear of COVID-19; Fear of the consequences (including death) determined by the COVID-19 is a significant predictor for the Need for emotional support; Work inappropriate conditions, despite their important potential, do not have much influence on the Need for emotional support. In addition to the direct effects mentioned above, some indirect effects of the Work conditions and Need for emotional support on burnout were highlighted. Work conditions have also indirect effects on burnout, mediated by the Fear of COVID-19. So, as predictor, Work conditions acts dually on the burnout syndrome, both directly and indirectly, through the Fear of the COVID-19 consequences, generating burnout and maintaining a strong sense of fear. Another indirect effect of Work conditions as a predictor of burnout is manifested through the Need for emotional support, but less significant; the Need for support, as a direct predictor of the burnout syndrome, proved to be a factor of little importance, but it could act as a blocking mechanism, inhibiting the direct effect of Work conditions on the burnout syndrome. The indirect inhibitory effect of the Need for emotional support as mediator between the Fear of COVID-19 and Burnout explains why the occurrence of the burnout syndrome is higher if the Need for emotional support is associated simultaneously with the inappropriate Work conditions and Fear of COVID-19.

In addition to the mediators of the burnout syndrome, Meaning of work was demonstrated to act as a moderating factor of these mediated relationships, due to the fact, that, as a direct predictor, Meaning of work showed negative effects on Burnout. The more the participants emphasize in their personal philosophy the importance of their role in ensuring public health, the more blocked the effects of the other predictors are. Also, even proved to be weak, the direct predictor role of Meaning of work on the Fear of the consequences (including death) determined by the COVID-19, was found to be inhibitory. Even if Work inappropriate conditions are a strong predictor of the Fear of COVID-19, this relationship is moderated by the Meaning of work. For this reason, the presence of a high level Meaning of work will reduce the negative effect of Work inappropriate conditions on Fear of infection. Meaning of life could not be included as burnout predictor due to its insignificant effective role at the Romanian national organizational level. In this context, the public health leaders and managers should implement organizational strategies to motivate employees by recognizing the high significance of the medical work, Meaning of work being both a predictive and a buffering determining factor in the burnout development.

The direct and active involvement of the institutional local or national leaders of the medical systems is mandatory aiming to provide essential and effective changes of the conditions that generate the increase of the burnout syndrome in any pandemic conditions. Future public health policies should include the involvement of the leaders of the healthcare system, both as effective helpers for healthcare workers, and providers of specific procedures.

The value of this study lies in the significant number of participants belonging to all categories of healthcare workers from all public medical entities in a university medical center in Romania which were involved in this research. By providing useful information for the managerial targeted approach of the burnout syndrome (as a specific negative effect of the COVID-19 pandemic), this study could open the gates to insure effective management procedures for other pandemic viral infections in the future.

## Limitations and Future Research Directions

This study has limitations such as the difficulty of distributing the questionnaires in a hard form and collecting data, during the lockdown. Analyzing data only for Romanian healthcare workers does not allow the full extrapolation of the results obtained for other health systems in the world. Another limitation could be the high heterogeneity of the study variables and population. A future study should focus on differences between medical personnel categories concerning burnout and its predictive factors. The internal validity of our study could also be affected by the recall bias. Individual experiences related to COVID 19 as healthcare worker or patient vary and recall of information depends entirely on memory which can often be imperfect and thereby unreliable; this bias could increase or decrease the strength of the observed associations. However, the data was collected during the pandemic, when participants faced the COVID 19 pandemic, which could limit the negative effect of recall bias.

As future research directions, further detailed analysis concerning the perceptions of the healthcare workers who were infected with SARS CoV-2 could provide relevant information to propose efficient interventions related to the burnout management. At the same time, creating and promoting guidelines including specific psychological interventions for the direct benefit of the healthcare workers involved in the care of patients with COVID-19 would be a relevant direction (68).

## Data Availability Statement

The raw data supporting the conclusions of this article will be made available by the authors, without undue reservation.

## Ethics Statement

The studies involving human participants were reviewed and approved by Council of the Faculty of Psychology and Education Sciences, Transilvania University of Brasov. The patients/participants provided their written informed consent to participate in this study.

## Author Contributions

SG and DG developed the study concept, conducted the data collection, and drafted the manuscript. A-MC performed the data analysis. LR conducted the literature review. All authors took part in result interpretation. All authors reviewed and edited several versions of the manuscript and provided critical revisions. All authors approved the final version of the manuscript for submission.

## Conflict of Interest

The authors declare that the research was conducted in the absence of any commercial or financial relationships that could be construed as a potential conflict of interest.

## Publisher's Note

All claims expressed in this article are solely those of the authors and do not necessarily represent those of their affiliated organizations, or those of the publisher, the editors and the reviewers. Any product that may be evaluated in this article, or claim that may be made by its manufacturer, is not guaranteed or endorsed by the publisher.
